# Patient participation in defining best-practice rheumatology service provision in Aotearoa New Zealand: a qualitative study with service consumers

**DOI:** 10.1186/s41927-022-00319-2

**Published:** 2023-01-24

**Authors:** Rachel Ngan Kee, Valerie Milne, Nicola Dalbeth, Rebecca Grainger

**Affiliations:** 1 Department of Medicine, University of Otago Wellington, 23a Mein St, PO Box 7343, Newtown, Wellington South 6242 New Zealand; 2 Patient Research Partner, Wellington, New Zealand; 3 Department of Medicine, Faculty of Medicine and Health Sciences, University of Auckland, Auckland, New Zealand; 4 Department of Rheumatology, Auckland District Health Board, Auckland, New Zealand; 5 Wellington Regional Rheumatology Unit, Hutt Valley District Health Board, Lower Hutt, New Zealand

**Keywords:** Patient and public involvement, Rheumatology, Health services

## Abstract

**Background:**

Aotearoa New Zealand (AoNZ) has no agreed models for rheumatology service provision in government-funded health care. We aimed to describe what people with inflammatory rheumatic diseases who have used rheumatology services view as being important in those services, and map these views to previously collated statements describing best practice components of rheumatology services from international recommendations. If these statements did not capture all service aspects that people with inflammatory rheumatic diseases considered important, we aimed to co-create new statements with our patient-participants.

**Methods:**

We conducted one focus group and an interview with people with inflammatory rheumatic disease who had used a government-funded rheumatology service in the previous 5 years (patient-participants) and analysed data using thematic analysis. The research team mapped subthemes to previously collated best practice recommendations that had been included in a Delphi consensus exercise with rheumatologists in AoNZ and proposed new statements, based on patient-participant data. Patient-participant feedback on thematic analysis and the new statements led to a refining of statements. A patient-partner in the research team informed research design and data analysis.

**Results:**

Patient-participants viewed it as highly valuable for rheumatology services to respect and value their experiences as people and patients, and those of their whānau (Māori word for family). They expected rheumatology services to provide the right care, at the right time. Many of the subthemes mapped to the best-practice statements. However, three new principles and three new statements were developed and refined by patient-participants. The three principles addressed valuing individuals, and their whānau (family) and their experiences, and providing a patient-focused health system that supports patient participation in decision-making and self-management, and patient education. New statements related to having a specific rheumatologist and other staff for comprehensive care, having adequate nurse staffing, and active provision of outside services and support.

**Conclusion:**

It was important to patients that rheumatology services demonstrated that patients and their whānau (family) were valued. The inclusion of people with rheumatic diseases who are users of rheumatology services in service development can provide valuable insights to inform how services should be delivered.

**Supplementary Information:**

The online version contains supplementary material available at 10.1186/s41927-022-00319-2.

## Background

Inflammatory rheumatic diseases and musculoskeletal diseases are an important and increasing cause of morbidity worldwide [1–6]. In Aotearoa New Zealand (AoNZ), estimates in 2018 suggested almost one in five New Zealanders aged 15 or over were living with at least one type of arthritis [2]. While all people with arthritis and musculoskeletal disease deserve appropriate health care to optimise health outcomes, people with inflammatory rheumatic diseases need specialist input from a rheumatologist, and an associated rheumatology health care team, for best health outcomes [7–11]. Although individually many inflammatory rheumatic diseases have a low prevalence, these diseases have high individual health burdens, with negative direct health outcomes, reduced quality of life, impacts on function, and high co-morbidity burden [12–15]. Thus, health systems need to provide appropriate specialist rheumatology services for people with inflammatory rheumatic diseases in order to achieve optimal health outcomes.

In AoNZ, most people with inflammatory rheumatic diseases access rheumatology services at no direct cost through government-funded district health boards (DHBs), with some care provided by rheumatologists in private practice in a fee-for-service model [1]. Initial access is via referral to these secondary services from general practitioners, or from other specialist services [1, 16]. For the last 20 years, the full-time equivalent (FTE) rheumatologist workforce in AoNZ has been consistently well below a recommended threshold of one per 60,000–80,000 population [1, 16, 17] which effectively limits access to rheumatology services. However, in addition to adequate FTE rheumatologists, international recommendations for best practice rheumatology services emphasise processes for care and the integral role of nurses and allied health professionals [18–23]. At present in AoNZ, there are no nationally agreed descriptions of components of government-funded rheumatology services. As an initial step in developing a national rheumatology service model for AoNZ, a Delphi exercise was undertaken to establish rheumatologists’ views on best practice rheumatology service components for rheumatology services in AoNZ. These were based on components previously proposed internationally. Of 22 statements offered, 16 statements reached consensus as being essential [24]. Further development of a rheumatology service model then required input from consumers of rheumatology services in AoNZ. The study described in this paper aimed to gain understanding of patients’ perspectives of public rheumatology services in AoNZ to inform this service model.

Patient-centred research aims to improve understanding, respect and shared commitment between patients and researchers, and ensure patient benefits of the research are not restricted to researcher and clinician goals but inclusive of patients, carers and the general public [25]. This cannot be meaningfully achieved without patient involvement [26, 27]. The benefits of patient involvement in health research in general are well-documented, including achieving more relevant outputs, improving patient recruitment and retention, reducing research waste produced by misalignment between research aims and end-user needs and enhancing research quality while empowering patients in the process [26, 28–30]. Health services research which incorporates patients often involves exploration of the emotional journey patients experience on their care pathway, creating space for understanding these experiences in order to improve patient care [31, 32]. Including patient perspectives of what rheumatology services should include is therefore fundamental to service delivery improvements that may support better patient experiences and care [31, 33].

It is vital that AoNZ develops a national rheumatology service model that can be used for benchmarking and ongoing quality evaluation. While rheumatologists’ views of potential service components were identified in the Delphi consensus exercise [24] we sought to understand what users of government-funded rheumatology services in DHB, people with inflammatory rheumatic diseases, viewed as being important components of those rheumatology services. This research was undertaken with patient involvement where this is defined as being carried out with or by patients rather than to, or about, or for them [34]. In this study, we aimed to describe what people with inflammatory rheumatic diseases in AoNZ who have used government-funded DHB rheumatology services view as important in those services. Additionally, we aimed to evaluate if these patient views were captured in the current rheumatologist-endorsed rheumatology service description statements, and if not, to co-create additional statements to express patient views.

## Methods

### Participants

Patient-participants were purposively recruited by Arthritis New Zealand from a database of people who had previously indicated interest in participation in research. Arthritis New Zealand is a non-governmental charitable organisation that provides information, advice, and support to people diagnosed with arthritis. Potential patient-participants were given a brief outline of the study and eligibility criteria. Inclusion criteria were any person in AoNZ who had used rheumatology services in any of the 20 DHB in the last 5 years, from 1st January 2016 to 30th November 2021. Service use was defined as ‘attendance in person at a DHB rheumatology clinic or associated allied health service, or participation in a video or telephone call with a rheumatologist or healthcare professional who works within a DHB rheumatology service’. Arthritis New Zealand aimed to recruit participants with a range of inflammatory rheumatic diseases, both sexes, and different ages and locations in AoNZ. A patient-participant contact list was provided to the research team by the research manager at Arthritis New Zealand and all nine potential patient-participants consented to participate.

Patient-participants were contacted by email by the research assistant, and provided with the information sheet. Informed consent was obtained and either a zoom-based focus group or interview then arranged.

### The research team

The research assistant was RNK (BHealSc), a female postgraduate medical student who was trained in interview skills by senior author, RG. RG (MBChB, PhD) is an academic rheumatologist with more than 10 years’ experience in qualitative research. ND (MBChB, MD) is an academic rheumatologist who is President of the New Zealand Rheumatology Association. VM (PhD) has a doctoral degree in health services research and lives with an inflammatory rheumatic disease. Participants were verbally informed that the study was initiated by RG and ND with a research goal of informing improvements in rheumatology service delivery in AoNZ and that RNK was employed to undertake the research under supervision. The dual role of researcher and service provider was explicitly considered by RG and ND at all stages of the research process, to acknowledge potential bias due to dual roles [35].

### Data collection

Data were collected in one focus group conducted via Zoom (interviewers RNK and RG) and an interview of a single participant via Zoom (interviewer RNK).

The same interview schedule was used for both the focus group and interview. This was developed by the research team and had four open-ended questions to elicit patient-participant perspectives of current rheumatology services and specific areas for improvement. These questions were (also Additional file 3: Interview Schedule):

1. What services do you value to support you in the management of your long-term arthritis/rheumatology condition?
2. What are the good aspects of DHB rheumatology services you have experienced?
3. What are the areas for improvement in the DHB rheumatology services you have experienced?
4. Describe your ideal DHB rheumatology service.

The 22 best-practice statements from the rheumatologist Delphi were edited or annotated to clarify terms used to increase accessibility for a lay audience by the research assistant (RNK) and the patient-research partner (VM) and then reviewed by the whole team. These were also presented to patient-participants for comment.

The focus group started with whakawhānaungatanga (Māori value of “developing connections”) including introductions and some informal conversation. Each open-ended question was then viewed using Google Slides (Google, LLC, 2022) with discussion facilitated. Screen-sharing was minimised to encourage conversation, with questions displayed in the Zoom (Zoom video communications, 2022) ‘Chat’ window. After the four questions, the 22 best practice statements used in the Delphi were each viewed and briefly discussed. This allowed patient-participants the opportunity to familiarise themselves with the statements and prepare for future review of any newly developed patient-participant data-derived statements. The focus group was concluded after 90 min as no new ideas were being offered (informational redundancy) and patient-participants confirmed they were satisfied they had opportunity to express all their thoughts and views. Researchers made field notes immediately after the focus group and interview. The focus group discussion and the interview were transcribed with Otter.ai (Otter a.i., 2022) then exported into Microsoft Word (Microsoft, 2022), with the recordings used to confirm accuracy of transcription.

### Data analysis

A thematic analysis using Braun and Clarke’s [36] six-phase approach was used, and specifically adopted an inductive approach. An inductive analysis was used as this maintains clear links between the research objectives and the findings derived from the data and ensures that these links are both transparent and defensible [37]. The six steps of analysis included; familiarisation with the data, generating initial codes, searching for patterns to develop themes, reviewing themes, defining and giving names to themes, and then summarising the themes in a report [38, 39]. The research assistant (RNK) led the analysis, with the senior author (RG) reviewing each phase of the analysis with the research assistant, particularly in finalising codes and themes. By two authors working together the quality and rigor of the analysis in development of the three themes was maintained [40].

Themes, subthemes and codes were then mapped to the 22 statements from the rheumatologist Delphi by two members of the research team (RNK and RG) by discussion and consensus. For the subthemes and codes that did not map to any of the 22 statements, the quotes linked to the codes were read and re-read to ensure understanding of patient-participant views. New statements about rheumatology services provision, directly informed by the raw data, were then drafted to represent the views underpinning the unmapped subthemes and codes.

### Patient-participant checking

We used member checking of synthesized analysed data as this is appropriate for both confirmation and co-construction [38, 39]. Patient-participants were emailed the data analysis report and the new statements developed from their patient-participant data to review. The first table presented the themes, subthemes and codes, along with representative quotes. A second table showed the mapping of themes, subthemes, and codes from patient-participant data to the 22 Delphi statements, the gaps, and new researcher-generated draft patient-data derived statements of rheumatology service components. An email was sent to patient-participants that invited them to reply with feedback, comments and suggested edits, and with a request for replies within a one-week timeframe.

Reporting has followed Consolidated criteria for reporting qualitative research (COREQ guidelines) [40] (checklist provided in Additional file 1) and Guidance for reporting of patient and public involvement (GRIPP 2) [34] (Checklist provided Additional file 2).

### Ethical considerations

The study was approved by the University of Otago Human ethics (Health) committee (H21/166) and endorsed by the Research Advisory Group (Māori) at the senior author’s DHB of employment (RAG-M #907). Written informed consent was obtained from all participants before the focus group and interview.

## Results

Of the nine patient-participants that were recruited, eight participants were included in the one focus group that was arranged at a time suitable to those participants (90 min long, interviewers RNK and RG). One participant was not available at the time of the focus group so was interviewed separately at a time more convenient to them (90 min long, interviewer RNK). Participant ages ranged from 47 to 71 years. Seven of the participants identified as female, and two as male. Six participants identified as New Zealand European/Pākehā, one as Māori (Indigenous New Zealander) and one as Māori and Samoan. Participants had a range of conditions for which they were accessing rheumatology services. These were reported by participants as; rheumatoid arthritis (five participants), “rheumatoid arthritis with connective tissue disorder, Raynaud’s and osteoporosis” (one participant), ankylosing spondylitis (one participant), “B27-related seronegative arthritis, ankylosing spondylitis and osteoarthritis” (one participant), “undifferentiated inflammatory arthritis” (one participant), and “systemic lupus erythematous with secondary Sjogren’s syndrome” (one participant). Participants came from seven different DHB across the North and South islands of AoNZ.

The codes from the thematic analysis were organised into two themes, and five sub-themes about two aspects of rheumatology service delivery. Themes with additional illustrative quotes are provided in Tables 1 and 2.

**Table 1:** Themes and subthemes for Theme 1 Experiences of individuals and whānau (family) are respected and valued

Sub-theme		Illustrative quote
*A1 Experience as people*		
A1.1	Value individuals and their experiences	
A1.1a	System	So that the person in the middle, the patient, is actually in the middle… We’re whole people. I’m not just my arthritis
A1.1b	Interpersonal	The main thing that I value personally is being respected as a person not just being a number and having someone that kind of understands what I’m going through
A1.2	Relationship with professionals is supportive	Everybody that I’ve come in contact with has been really supportive
A1.3	Importance of relationships with professionals in the rheumatology service	
A1.3a	Rheumatologists	Well, I’m hoping [my rheumatologist is] not going anywhere. I’ve finally found my rapport, he checked all my boxes when I had my many questions when I saw him for the first time
A1.3b	Nurses	And his nurse has been awesome
A1.3c	Support/admin staff organising appointments	Establishing a relationship with both the specialty nurse and whoever’s in the rheumatology department and your rheumatologist is pivotal
*A2 Experiencing care that supports the patient*		
A2.1	Supporting self-management	And learning how to manage all of that yourself is really empowering
A2.2	Supporting active participation in decision-making and care	There’s an obligation on the patient to actually try really hard and try and learn their condition so that they can be a part of that conversation and not be a passive adverse observer and be someone who things happen to
A2.3	Education requirements met	
A2.3a	Right language for effective communication	How to be an effective patient and how to communicate effectively, and the importance of knowing the language of your condition so you’re not flapping around in the dark
A2.3b	About condition and management	A lot more education about what was wrong but also about what services are available and how to access them as well
A2.3c	To make informed decisions	It will be very helpful to have information prior to seeing the rheumatologist so you’re able to make an informed decision

**Table 2:** Themes and subthemes for theme 2 right care, right time

Sub-theme		Illustrative quote
*B1 Rheumatology specialist care*		
B1.1	Sufficient rheumatologists	If you see a rheumatologist it’s like gold… It’s very difficult to see a rheumatologist
B1.2	Timeliness	
B1.2a	Of diagnosis	I’m very grateful for his diagnosis on the day. So very different from nowadays where you can be diagnosed with methotrexate on day one
B1.2b	Appointments (including appointment certainty)	I see a really stretched health system with really stretched Allied Health, long wait lists, and services that are difficult to access
B1.3	Appropriately responsive care access mechanisms	If you’re complex and you need it, then you can get that care that you need
B1.4	Patient-factors related to access	
B1.4a	Telehealth	There’s no, there’s no travel involved. And, and for a lot of us who have chronic fatigue and have pain, that that travel comes at a human cost and if you’re on a benefit that comes at a financial cost, which sometimes is onerous if you’ve got you know, a run of them
B1.4b	Funding	Getting the physiotherapy or occupational therapy has been non-existent effectively, I’ve had to do all that myself at my own expense
B1.4c	Mobility pass	–
*B2: Access to rheumatology nurses is highly important*		
B2.1	Between appointments	The nurse, who works in conjunction with [the rheumatologist] is [who] we can contact if we need assistance appointments
B2.2	Nurse phone line	Having the access to the rheumatology nurses as often, you know, once a week or anything, I always felt I could ring and that was lovely
*B3: Co-ordinated care and other aspects of care*		
B3.1	Coordinated care	
B3.1a	Between specialists	Communication between orthopaedics and rheumatology is just brilliant
B3.1b	Between DHBs	I’m looking forward to the day when all DHB records are interchangeable and easily accessed because they’ve created hiccups in my care
B3.1c	Across disciplines (non-specialist)	When your disease is compromising your whole system, then you end up having to cross lots of different specialties, but also lots of different services
B3.2	Allied health	
B3.2a	Physiotherapy	The other really good thing that I value about rheumatology is the rheumatology service works really close with physios in the hydrotherapy pool. So there’s a wider range of treatment services that could be available to you and they support your rehab and things like that
B3.2b	Occupational therapy	I tried really hard to get access to occupational health therapy and couldn’t
B3.2c	Personal Trainer	Cos a big thing I’ve found with exercise is I have to have someone else there to motivate me to go along, which is why I was seeing a personal trainer
B3.2d	Carer	Just recently I had a full time carer look after me for the last 7 months, but was told two Fridays ago that she wasn’t available
B3.2e	Orthotics/podiatrist	Just, really there’s been no hesitation from [the rheumatologist] I saw in October I’ve seen the ortho pro guy, lovely
B3.2f	Pharmacy	You’ll be contacted by a rheumatologist nurse, you’ll be able to pick up a script, just give us time to get it all printed, sent directly to the chemist. That would be very helpful
B3.2 g	Oral hygienist	To see a hygienist every 4 months because of my hands
B3.3	Access to specialist multidisciplinary care when relevant	
B3.3a	Pain	Chronic health occupational therapist was her title, and she had worked in the pain management clinic in Wellington. And what she did was run sort of, pain management clinic for people with chronic health conditions … Able to bring in, you know, like a physio and the rheumatology nurse and you know, and get a whole group of people, different sorts of providers together to be able to workshop for the patient
B3.4	System navigation	It would be quite helpful to know what the actual process is. If I was a new person diagnosed with arthritis. How often, how long would you normally have to wait to see a rheumatologist and what support you are given after you’ve been diagnosed?
B3.4a	Support groups	If people who are newly diagnosed were given the contact details of… of these various support agencies that would support them. there’s a huge amont of support groups in all areas of New Zealand, both online and face to face and I think those… those groups, enable people to learn how to live with the condition and… and nor… and help normalize it especially when you’re newly diagnosed
B3.4b	Other services	–
B3.5	Access to personal health information	Learning the importance of things like manage my health and knowing to ask for copies of letters going to and fro, and keeping those and knowing your test results and what those tests result mean for your particular condition. Being able to trot off what meds you’re on and why, and you know what milligrams they are and all of that stuff

### Theme 1: Experiences of individuals and whānau (family) are respected and valued

Most patient-participants regarded feeling respected as people in the role of patient as being highly important when using DHB rheumatology services. They also valued the same respectful interaction with their whānau (Māori word for family, in common use in AoNZ). For many, feeling as though they were valued for who they are was the difference between their experience of the rheumatology service considered positive or negative. This theme’s two sub-themes were; experience as people, and experiencing care that supports the patient.

#### Experience as people

Patient-participants stated the importance of being valued and respected as people, noting this was important at both the system level as well as at the interpersonal level. At the system level, patient-participants wanted services to be accommodating of the common day-to-day challenges for people with inflammatory rheumatic diseases, such as managing pain, accessibility barriers for transport and buildings, and managing interactions with multiple healthcare specialties and providers. A participant expressed this core value as being akin to principles of patient-centred care [41, 42].

So that the person in the middle, the patient, is actually in the middle… We’re whole people. I’m not just my arthritis. – Participant, 48years

At the interpersonal level, participants experienced rheumatology care as positive when healthcare professionals were empathetic, available, and engaging. Correct name pronunciation, having a comfortable relationship and feeling as though their healthcare professional genuinely cared were offered as examples of positive interpersonal interactions.

*T*he main thing that I value personally is being respected as a person not just being a number and having someone that kind of understands what I’m going through. – Participant, 56years

Participants also highly valued the personal relationship with their rheumatologist.

Well, I’m hoping [my rheumatologist is] not going anywhere. I’ve finally found my rapport, he checked all my boxes when I had my many questions when I saw him for the first time. – Participant, 53years

The importance of relationships with professionals in the rheumatology service was highlighted to extend beyond rheumatologists to the healthcare team, particularly rheumatology nurses and support and administration staff.

Establishing a relationship with both the specialty nurse and whoever’s in the rheumatology department and your rheumatologist is pivotal. – Participant, 69years

#### Experiencing care that supports the patient

Patient-participants wanted care that acknowledged their lived experiences as a person with a chronic disease. Participants wanted support for their self-management, noting that in their role as patients they need to know how to manage their condition as independently as possible.

There’s an obligation on the patient to actually try really hard and try and learn their condition so that they can be a part of that conversation and not be a passive adverse observer and be someone who things happen to. – Participant, 69years

Patient-participants viewed information provision as an important way that services and healthcare professionals can support their active participation in decision-making and care, including education about their condition and its management, and learning correct language for effective communication to support informed decision-making.

A lot more education about what was wrong but also about what services are available and how to access them as well. – Participant, 47years

### Theme 2: right care, right time

This theme was about receiving high-quality care at the right time, and from the appropriate healthcare professional. This theme’s three sub-themes were; appropriate access to rheumatologists, appropriate access to rheumatology nurses, and co-ordinated care and other aspects of care.

#### Appropriate access to rheumatologists

Patient-participants considered rheumatologists essential for timely diagnosis. They also noted a quality rheumatology service provided appointments with rheumatologists at appropriate intervals and provided some certainty that this would occur. The current shortage of rheumatologists was specifically mentioned by several participants, and many made the link between this and inappropriately long appointment wait-time.

If you see a rheumatologist it’s like gold… It’s very difficult to see a rheumatologist. – Participant, 53years

Patient-participants considered a high quality rheumatology service would have mechanism to provide rheumatologist appointments urgently, or at short notice, if clinically needed.

If you’re complex and you need it, then you can get that care that you need. – Participant, 48years

Additionally, patient factors related to access would be considered and acknowledged in the planning of specialist rheumatologist appointments, such as flexibility to have appointments online if clinically appropriate and referral for relevant subsidies covering out-of-pocket costs of attending.

There’s no travel involved. And, for a lot of us who have chronic fatigue and have pain, that travel comes at a human cost and if you are on a benefit that comes at a financial cost, which is sometimes onerous if you’ve got a run of them. – Participant, 69years

#### Appropriate access to rheumatology nurses

Access to rheumatology nurses was considered very important by participants, with many stating these nurses were their primary contact for inflammatory rheumatic disease management between rheumatologist appointments. Participants valued being able to talk to a rheumatology nurse about any issues relating to their inflammatory rheumatic disease management.

The nurse, who works in conjunction with [the rheumatologist] is [who] we can contact if we need assistance appointments. – Participant, 71years

Nurses were seen as more accessible than rheumatologists. Many participants emphasized how much they valued first-hand experiences of phone-line availability for contacting nurses to support personal and practical management of their rheumatic disease.

Having the access to the rheumatology nurses as often, you know, once a week or anything, I always felt I could ring and that was lovely. – Participant, 67years

#### Co-ordinated care and other care

In addition, patient-participants valued co-ordinated care, allied health involvement, access to specialised multidisciplinary care when relevant, assistance with system navigation and access to personal health information as important factors of quality care. Patient-participants wanted to see co-ordinated care between specialists, between DHBs and to other non-specialist disciplines. Multidisciplinary care in specialised pain clinics was also highly valued by the participant that had experienced it.

Communication between orthopaedics and rheumatology is just brilliant. – Participant, 61years

Allied health involvement was noted as extremely important, particularly occupational therapy, physiotherapy, personal carers, orthotics/podiatrists, pharmacists and oral hygienists.

The other really good thing that I value about rheumatology is the rheumatology service works really close with physios in the hydrotherapy pool. So there’s a wider range of treatment services that could be available to you and they support your rehab and things like that. – Participant, 48years

Patient-participants considered rheumatology services could play a larger role in assisting patient navigation to other health care support, particularly to support groups, but also to other services that could assist them such as mobility passes and other accessibility aids.

[If] people who are newly diagnosed were given the contact details of these various support agencies that would support them. There’s a huge amount of support groups in all areas of New Zealand, both online and face to face and I think those groups, enable people to learn how to live with the condition and help normalize it, especially when you’re newly diagnosed. – Participant, 69years

Finally, patient-participants felt rheumatology services should provide easy and open access to their personal health information, to aid communication and movement through the health system.

### New statements from participant views after mapping to previously identified best-practice statements

Mapping of themes, subthemes and codes to best practice statements used in the rheumatologist Delphi showed that no subthemes or codes from Theme 1—“Experiences of individuals and whānau (family) are respected and valued”—were described by these statements (Table 3 shows mapping, Delphi statements provided in Additional file 4: Table S1). Three principles for rheumatology services were drafted based directly on patient-participant quotes from Theme 1 (Table 3). New statements related to having identified or named rheumatologist and other staff for care (Statement 23—from Theme 1, subtheme A1.3), having adequate nursing staffing (full-time equivalents) (statement 24—from Theme 2, subtheme B2) and active provision of outside services and support (statement 25—from Theme 2, subtheme B3.4a/b) (Table 2). There were 11 subthemes that did not map but were considered by the research team, within the context of standard health service arrangements in AoNZ, to be outside scope or influence of government-funded rheumatology services. These subthemes are more appropriately located within primary care or community service (n = 7), a whole of DHB issue (n = 3) or related to other DHB service availability (n = 1) (Table 3).

**Table 3:** Cross-referencing of Delphi best-practice rheumatology service statements to themes from patient data, and generation of new statements and overarching principles to themes without a match

Subthemes	Mapped to Delphi best practice statement (No or number of statement)	Statement or principle encompassing patient views derived from data
Value individuals and their experiences	No	New principle 1: A rheumatology service should value individuals and their experiences through positive interpersonal interactions, supportive relationships and within a health system organised with the patients’ needs at the centre
System	No	
Interpersonal	No	
Relationship with professionals is supportive	No	
Importance of relationships with professionals in the rheumatology service	No	New statement 23: Patients should have specific rheumatologist(s) responsible for their care and be provided with the names and roles of other medical, nursing, allied health and administrative staff who may be involved in their care
Rheumatologists	No	
Nurses	No	
Support/admin staff organising appointments	No	
Supporting self-management	No	New principle 2: Healthcare professionals in a rheumatology service actively support patients to participate in decision-making and self-management
Supporting active participation in decision-making and care	No	
Education requirements met	5	New principle 3: Healthcare professionals in a rheumatology service should ensure patients’ education requirements about their rheumatic condition are met; including appropriate communication, content, and framed to support patients’ active involvement in shared decision-making
Right language for effective communication	No	
About condition and management	No	
To make informed decisions	No	
Rheumatology specialist care	4, 16	
Timeliness	2, 3, 16	
Of diagnosis	2,3,16	
Appointments (including appointment certainty)	No	Carer—Outside of rheumatology—primary/community health provision
Appropriately responsive care access mechanisms	8, 16, 21	
Patient-factors related to access	20	
Telehealth	20	
Funding	No	Funding for allied health—Outside of rheumatology—primary/community health provision
Mobility pass	No	Mobility pass—Outside of rheumatology—primary/community health provision
Access to rheumatology nurses is highly important	3, 5, 6, 7, 8	NEW STATEMENT 24: A public rheumatology service should involve at least one full time equivalent (FTE) rheumatologist nurse per FTE rheumatologist
Between appointments	7	
Nurse phone line	6, 7	
		Access to Pain MDT—Outside of rheumatology—Other DHB service
Co-ordinated care and other aspects of care	15	
Between specialists	15	
Between DHBs	No	DHB/service communication Outside of rheumatology—whole of DHB
Across disciplines (non-specialist)	No	DHB/service communication Outside of rheumatology—whole of DHB
Allied health	9, 10, 11, 15	
Physiotherapy	11	
Occupational therapy	9	
Personal trainer	No	Personal trainer—Outside of rheumatology—primary/community health provision
Carer	No	Carer—Outside of rheumatology—primary/community health provision
Orthotics/podiatrist	10	
Pharmacy	No	Pharmacist—Outside of rheumatology—primary/community health provision
Oral hygienist	No	Dental hygienist—Outside of rheumatology—primary/community health provision
Access to specialist multidisciplinary care when relevant	13	
Pain	13	
System navigation	No	New statement 25: Rheumatology services should actively provide information to patients with rheumatic diseases about outside services or providers that provide social, emotional or practical support
Support groups	No	
Other services	No	
Access to personal health information	No	Access to personal health information—Outside of rheumatology—whole of DHB

Abbreviations: *DHB* District Health Board, *MDT* multidisciplinary team

Four patient-participants responded to the email about data analysis and new statements. Overall, the analysis and new principles and statements were endorsed by these participants, subject to some rewording for clarity, which was agreed upon and revised. All four patient-participants provided feedback that they viewed the objectives of the research and the process and their involvement very positively.

## Discussion

Patient views of important and valued aspects of best practice rheumatology services in AoNZ had two themes; services that value patient and whānau (family) experiences as patients and people, and providing “right care, right time” where appropriate rheumatologist, rheumatology nurse and other care can be accessed efficiently. Patients wanted to feel valued and respected as individuals, and have their experiences valued by both the system itself and through interpersonal interactions. They also wanted a service that provides them access to services they need, when they need it, and from the appropriate healthcare professional. Many of the themes mapped directly to the existing statements of components of best practice rheumatology service, however from those that did not map, three new principles of care and three new statements were created directly from patient views and endorsed by our patient-participants.

These findings explicitly call to attention the need for consideration of components beyond mechanisms of service delivery. There is relatively little literature on what patients in AoNZ value in healthcare services delivery or internationally what people with inflammatory rheumatic diseases value in rheumatology services. A previous study including people with inflammatory rheumatic diseases from a DHB rheumatology service in AoNZ reported how much these patients valued appointments with rheumatologists and rheumatology nurses [43]. A meta-synthesis of qualitative studies of experiences of Māori in health services in AoNZ also identified similar factors to those in our study as influencing positive or negative experiences of healthcare, finding positive health service experiences were facilitated by positive interactions, support navigating the health system and practical support, with barriers relating to negative staff interactions and organisational structures [44]. A synthesis of quantitative and qualitative literature also identified whakawhānaungatanga, whānau (family), and manaakitanga (kindness) as being key features facilitating engagement in health services for Māori [45]. Internationally, an implementation evaluation of group clinics in a rheumatology service network in the United Kingdom (UK) collected views of people with osteoporosis and inflammatory arthritis about the clinics [46]. Features that enabled this innovative care model were characterised in the themes of efficiency, empathy, education, engagement, and empowerment, with the promoting factors for implementation including appropriate prioritisation, personalization, and participation. These are remarkably similar ideas to those characterised as desirable by our patient-participants. Another focus group study in a rheumatology service in the UK also reports similar aspects of rheumatology care to be valued by patients and should continue to be improved; these included acknowledgement of factors of importance to the patient, patient-centred care and continuity of care [47]. Although the data are limited, these studies support our data in emphasising the importance of relationships and aspects of patient-centred care.

While delivery of high-quality services according to measurable process or quality metrics is key, our findings also emphasise the importance of patient experiences and how people are treated when accessing health services. Though this research was conducted in the context of public rheumatology services in AoNZ, patient-based qualitative methodological approaches to improving service delivery can be applied to any discipline, in any country. The overarching principles pertaining to Theme 1, such as attitudes of health systems and healthcare professionals within that system toward patients, should form the basis of all patient-centred care. Overall, our findings and these studies emphasise the high importance people in AoNZ put on positive and culturally appropriate interpersonal interactions with health care professionals and health services.

To our knowledge this is the first publication which has described the views of a group of patients with inflammatory rheumatic disease on positive aspects and areas of improvement for public rheumatology services in AoNZ. This work could not have been undertaken without the involvement of patients in the design of the research (patient-research partner) and in data collection. Additionally, focus group findings have been mapped against a set of best practice rheumatology service components that were previously compiled, and patient views have contributed further aspects of rheumatology care to be considered as best practice components. These data can inform future rheumatology service delivery from AoNZ DHB rheumatology services. This is very relevant and timely as AoNZ is due to begin nationwide health system reforms in July 2022. Such changes invite the possibility to improve on many aspects of healthcare service delivery, including the opportunity to completely re-design future public health services in a manner that is consistent with patient expectations. Our set of three principles and 25 statements of components of a best practice rheumatology service for AoNZ can be used to inform policy and service changes. We plan to collect data from a nationwide sample of people who have used DHB rheumatology services to evaluate their agreement, or otherwise, with each of the statements we have compiled from international literature [24] and this study. In this way, we hope to provide further understandings of what people with inflammatory rheumatic diseases value and consider most important in DHB rheumatology services. Patient-participants in this project had very positive views of the intent of the research—to involve patients in determining important aspects of health care delivery. The research team experienced a sense of humility and responsibility in undertaking this work. Overall, the research team and patient-participants found this a highly positive experience.

Our study has limitations. Our patient-participants were purposively recruited and varied in age, DHB region and inflammatory rheumatic disease diagnosis but cannot be considered to represent views of all people with inflammatory rheumatic disease in AoNZ. Using a focus group does enable a diversity of views to be elicited, with all participants supported to engage [48]. The data collection by focus group and an interview included all patient participants offered by Arthritis New Zealand. We viewed that since we had appeared to reach informational redundancy and that the data collection was to extend a current set of statements, and not generate a theory or statements de novo, the sample size was adequate for our purposes [49]. While it is possible that a larger focus group or multiple focus groups may have elicited additional ideas, this study offers the first a literature- and patient-informed descriptors rheumatology services in AoNZ as a foundation to service development. An in-person focus group, which may have generated different discussion, was not feasible due to practical aspects of COVID-19 limiting desire to travel. In-person group discussions may involve secondary conversations within the main discussion that can contribute richly to findings. Undertaking member checking and review of new principles and statements via email may have limited participant ability to fully discuss any changes. Our study has been undertaken in AoNZ in an inflammatory rheumatic disease patient population and the findings might not be widely generalizable outside of AoNZ or in other patient populations in AoNZ.

## Conclusion

This study elicited patient views of positive aspects of current and future rheumatology services in AoNZ for the first time. With these views, combined with the previously collated statements about best practice rheumatology care, we now have a basis for further engagement with patients, rheumatologists, rheumatology nurses and allied health, DHB service providers and government policy makers and funders to shape future rheumatology services. Importantly, patients highlighted that their treatment and interpersonal interactions in the health system, has a large impact on their experience. This signals a need for health systems to place more emphasis on overarching principles of care that includes positive interpersonal communication and being empathetic to people’s experiences as patients, not just mechanisms of service delivery.

## Supplementary Information

**Additional file1**. COREQ (COnsolidated criteria for REporting Qualitative research) Checklist.

**Additional file2**. **Table 1**: GRIPP2 long form.

**Additional file3**. Interview Schedule. The interview schedule for the focus group and interview.

**Additional file4**. Table S1. Statements used in Delphi exercise with rheumatologists describing potential components of a best-practice rheumatology service in DHB in AoNZ [24].

## Data Availability

The corresponding author will consider any reasonable request for the qualitative data set.

## Abbreviations

AoNZ: Aotearoa New Zealand
COREQ: Consolidated criteria for reporting qualitative research
COVID-19: Novel coronavirus disease 2019
DHB: District health boards
FTE: Full time equivalent
GRIPP 2: Guidance for reporting of patient and public involvement
UK: United Kingdom

## References

[1] Harrison AA, Tugnet N, Taylor WJ (2019). A survey of the New Zealand rheumatology workforce. N Z Med J.

[2] Deloitte Access Economics. The economic cost of arthritis in New Zealand in 2018 [Internet]. Arthritis New Zealand; 2018 [cited 2021 Nov 29] p. 93. Available from: https://www.arthritis.org.nz/wp-content/uploads/Economic-Cost-of-Arthritis-in-New-Zealand-2018.pdf.

[3] Cross M, Smith E, Hoy D, Nolte S, Ackerman I, Fransen M (2014). The global burden of hip and knee osteoarthritis: estimates from the global burden of disease 2010 study. Ann Rheum Dis.

[4] Cross M, Smith E, Hoy D, Carmona L, Wolfe F, Vos T (2014). The global burden of rheumatoid arthritis: estimates from the global burden of disease 2010 study. Ann Rheum Dis.

[5] Smith E, Hoy D, Cross M, Merriman TR, Vos T, Buchbinder R (2014). The global burden of gout: estimates from the global burden of disease 2010 study. Ann Rheum Dis.

[6] Smith E, Hoy DG, Cross M, Vos T, Naghavi M, Buchbinder R (2014). The global burden of other musculoskeletal disorders: estimates from the global burden of disease 2010 study. Ann Rheum Dis.

[7] Smolen JS, Landewé RBM, Bijlsma JWJ, Burmester GR, Dougados M, Kerschbaumer A (2020). EULAR recommendations for the management of rheumatoid arthritis with synthetic and biological disease-modifying antirheumatic drugs: 2019 update. Ann Rheum Dis.

[8] Gossec L, Smolen JS, Ramiro S, de Wit M, Cutolo M, Dougados M (2016). European league against rheumatism (EULAR) recommendations for the management of psoriatic arthritis with pharmacological therapies: 2015 update. Ann Rheum Dis.

[9] Gossec L, Baraliakos X, Kerschbaumer A, de Wit M, McInnes I, Dougados M (2020). EULAR recommendations for the management of psoriatic arthritis with pharmacological therapies: 2019 update. Ann Rheum Dis.

[10] Fanouriakis A, Kostopoulou M, Alunno A, Aringer M, Bajema I, Boletis JN (2019). 2019 update of the EULAR recommendations for the management of systemic lupus erythematosus. Ann Rheum Dis.

[11] Mackie SL, Dejaco C, Appenzeller S, Camellino D, Duftner C, Gonzalez-Chiappe S (2020). British Society for rheumatology guideline on diagnosis and treatment of giant cell arteritis. Rheumatology.

[12] Safiri S, Kolahi AA, Hoy D, Smith E, Bettampadi D, Mansournia MA (2019). Global, regional and national burden of rheumatoid arthritis 1990–2017: a systematic analysis of the Global Burden of Disease study 2017. Ann Rheum Dis.

[13] Boehncke WH, Menter A (2013). Burden of disease: psoriasis and psoriatic arthritis. Am J Clin Dermatol.

[14] Boonen A, van der Linden SM (2006). The burden of ankylosing spondylitis. J Rheumatol Suppl.

[15] Carter EE, Barr SG, Clarke AE (2016). The global burden of SLE: prevalence, health disparities and socioeconomic impact. Nat Rev Rheumatol.

[16] Harrison A (2004). Provision of rheumatology services in New Zealand. N Z Med J.

[17] British Society for Rheumatology: BSR. Rheumatology workforce: a crisis in numbers [Internet]. British Society for Rheumatology (BSR); 2021 [cited 2021 Nov 29]. Available from: https://rheumatology.org.uk/Portals/0/Documents/Policy/Reports/BSR-workforce-report-crisis-numbers.pdf.

[18] National Institute for Health and Care Excellence. Rheumatoid arthritis in adults: management [Internet]. 2018 [cited 2022 Mar 8]. Available from: www.nice.org.uk/guidance/ng100.

[19] Rheumatoid. Arthritis in over 16’s. Quality Standard.

[20] Cooper M, Rouhi A, Barber CEH (2018). A systematic review of quality measures for inflammatory arthritis. J Rheumatol.

[21] EULAR | Recommendations. and initiatives EULAR/acr [Internet]. [cited 2022 Mar 8]. Available from: https://www.eular.org/recommendations_eular_acr.cfm.

[22] Clinical Practice Guidelines [Internet]. [cited 2022 Mar 8]. Available from: https://www.rheumatology.org/Practice-Quality/Clinical-Support/Clinical-Practice-Guidelines.

[23] Guidelines | British Society for Rheumatology [Internet]. [cited 2022 Mar 8]. Available from: https://www.rheumatology.org.uk/practice-quality/guidelines.

[24] Gibbs H, Grainger R (2022). A Delphi exercise with rheumatologists to identify consensus on essential components of a rheumatology service in District Health Boards of Aotearoa New Zealand. NZMJ.

[25] Boote J, Wong R, Booth A (2012). ‘Talking the talk or walking the walk?’ A bibliometric review of the literature on public involvement in health research published between 1995 and 2009. Health Expect.

[26] Sacristán JA, Aguarón A, Avendaño-Solá C, Garrido P, Carrión J, Gutiérrez A (2016). Patient involvement in clinical research: why, when, and how. Patient Prefer Adherence.

[27] Tinetti ME, Basch E (2013). Patients’ responsibility to participate in decision making and research. JAMA.

[28] Slattery P, Saeri AK, Bragge P (2020). Research co-design in health: a rapid overview of reviews. Health Res Policy Syst.

[29] Domecq JP, Prutsky G, Elraiyah T, Wang Z, Nabhan M, Shippee N (2014). Patient engagement in research: a systematic review. BMC Health Serv Res.

[30] Schilling I, Behrens H, Hugenschmidt C, Liedtke J, Schmiemann G, Gerhardus A (2019). Patient involvement in clinical trials: motivation and expectations differ between patients and researchers involved in a trial on urinary tract infections. Res Involv Engagem.

[31] Boyd H, McKernon S, Mullin B, Old A (2012). Improving healthcare through the use of co-design. N Z Med J.

[32] van der Scheer L, Garcia E, van der Laan AL, van der Burg S, Boenink M (2017). The benefits of patient involvement for translational research. Health Care Anal.

[33] Maher L, Hayward B, Hayward P, Walsh C (2017). Increasing patient engagement in healthcare service design: a qualitative evaluation of a co-design programme in New Zealand. Patient Exp J.

[34] Staniszewska S, Brett J, Simera I, Seers K, Mockford C, Goodlad S (2017). GRIPP2 reporting checklists: tools to improve reporting of patient and public involvement in research. BMJ..

[35] Hay-Smith EJC, Brown M, Anderson L, Treharne GJ (2016). Once a clinician, always a clinician: a systematic review to develop a typology of clinician-researcher dual-role experiences in health research with patient-participants. BMC Med Res Methodol.

[36] Braun V, Clarke V (2022). Thematic analysis: a practical guide.

[37] Thomas DR (2006). A General inductive approach for analyzing qualitative evaluation data. Am J Eval.

[38] Harvey L (2015). Beyond member-checking: a dialogic approach to the research interview. Int J Res Method Edu.

[39] Birt L, Scott S, Cavers D, Campbell C, Walter F (2016). Member checking: a tool to enhance trustworthiness or merely a nod to validation?. Qual Health Res.

[40] Tong A, Sainsbury P, Craig J (2007). Consolidated criteria for reporting qualitative research (COREQ): a 32-item checklist for interviews and focus groups. Int J Qual Health Care.

[41] Institute of Medicine (US) Committee on Quality of Health Care in America. Crossing the Quality Chasm: A New Health System for the 21st Century [Internet]. Washington (DC): National Academies Press (US); 2001 [cited 2022 Mar 13]. Available from: http://www.ncbi.nlm.nih.gov/books/NBK222274/. 25057539

[42] Epstein RM, Street RL (2011). The values and value of patient-centered care. Ann Fam Med.

[43] Grainger R, Townsley HR, Ferguson CA, Riley FE, Langlotz T, Taylor WJ (2020). Patient and clinician views on an app for rheumatoid arthritis disease monitoring: function, implementation and implications. Int J Rheum Dis..

[44] Espiner E, Paine SJ, Weston M, Curtis E (2021). Barriers and facilitators for Māori in accessing hospital services in Aotearoa New Zealand. N Z Med J.

[45] Graham R, Masters-Awatere B (2020). Experiences of Māori of Aotearoa New Zealand’s public health system: a systematic review of two decades of published qualitative research. Aust New Zealand J Public Health.

[46] Russell-Westhead M, O’Brien N, Goff I, Coulson E, Pape J, Birrell F (2020). Mixed methods study of a new model of care for chronic disease: co-design and sustainable implementation of group consultations into clinical practice. Rheumatol Adv Pract.

[47] White KM, Ivan A, Williams R, Galloway JB, Norton S, Matcham F (2021). Remote measurement in rheumatoid arthritis: qualitative analysis of patient perspectives. JMIR Form Res..

[48] Powell RA, Single HM (1996). Focus groups. Int J Qual Health Care.

[49] Sandelowski M (1995). Sample size in qualitative research. Res Nurs Health.

