# Supplementary material for: Patient participation in defining best-practice rheumatology service provision in Aotearoa New Zealand: a qualitative study with service consumers
Source: BMC Rheumatol. 2023 Jan 24;7:1. doi: 10.1186/s41927-022-00319-2 (PMC9872402; doi:10.1186/s41927-022-00319-2)
Supplement: Supplementary file 3 — Additional file3. Interview Schedule. The interview schedule for the focus group and interview. [file 41927_2022_319_MOESM3_ESM.docx]

Interview Schedule

1. What services do you value to support you in the management of your long-term arthritis/rheumatology condition?
2. What are the good aspects of DHB rheumatology services you have experienced?
3. What are the areas for improvement in the DHB rheumatology services you have experienced?
4. Describe your ideal DHB rheumatology service.
