# Supplementary material for: Patient participation in defining best-practice rheumatology service provision in Aotearoa New Zealand: a qualitative study with service consumers
Source: BMC Rheumatol. 2023 Jan 24;7:1. doi: 10.1186/s41927-022-00319-2 (PMC9872402; doi:10.1186/s41927-022-00319-2)
Supplement: Supplementary file 4 — Additional file4. Table S1. Statements used in Delphi exercise with rheumatologists describing potential components of a best-practice rheumatology service in DHB in AoNZ [24]. [file 41927_2022_319_MOESM4_ESM.docx]

Supplemental Table 1. Statements used in Delphi exercise with rheumatologists describing potential components of a best-practice rheumatology service in DHB in AoNZ (24)

| Statement |
| --- |
| 1. Patients with active rheumatoid arthritis should be offered the opportunity to commence conventional disease-modifying anti-rheumatic drug (DMARD) therapy (e.g., methotrexate, sulfasalazine, hydroxychloroquine), within six weeks of referral to a rheumatology service. |
| 2. Patients with active rheumatoid arthritis should be monitored 3-monthly, using a composite score such as DAS-28 CRP/ESR, until their treatment target is met. |
| 3. Patients with chronic rheumatic disease should have access to a rheumatology service to support coordinating their care (e.g., with a rheumatology nurse specialist or rheumatologist). |
| 4. Patients with chronic rheumatic disease and disease flares, or possible treatment-related side effects, should receive advice within 1-working day of contacting a rheumatology service. |
| 5. Patients with chronic rheumatic disease should have access to a nurse for education. |
| 6. Patients with chronic rheumatic disease should have access to a nurse-led telephone service for ongoing support. |
| 7. Specialist rheumatology nurses should participate in comprehensive disease management of chronic rheumatic disease. |
| 8. Within an outpatient rheumatology clinic, a specialised rheumatology nurse should have their own consultations with chronic rheumatic disease patients. |
| 9. Patients with chronic rheumatic disease, and difficulties with activities of daily living (ADLs), or hand function, should have access to specialist occupational therapy, and/or hand therapy. |
| 10. Patients with chronic rheumatic disease and active foot problems should have access to podiatry assessment and ongoing review. |
| 11. Patients with chronic rheumatic disease should have access to specialist physiotherapy, with periodic review. |
| 12. A rheumatology service should have timely access to musculoskeletal imaging, including ultrasound and magnetic resonance imaging (MRI), to aid in the diagnosis and management of inflammatory arthritis. |
| 13. Patients with chronic rheumatic disease who suffer from pain issues, should have access to a qualified health professional who specialises in chronic pain management (e.g., specialist pain management physician or psychologist). |
| 14. A rheumatology service should include an infusion unit for the delivery of specialist-prescribed intravenous medications (e.g., infliximab, tocilizumab, rituximab), which is supervised (directly, or at a distance) by a member of the rheumatology service. |
| 15. A rheumatology service should aim to involve other specialists in "combined clinics", where the management of chronic disease spans across different specialties (e.g., combined clinics with dermatology or ophthalmology). |
| 16. A public rheumatology service should involve at least one full time equivalent (FTE) rheumatologist per 80,000 people within the served population. |
| 17. A rheumatology service should provide outpatient assessment for patients with non-inflammatory musculoskeletal conditions, such as fibromyalgia and osteoarthritis, when specialist input is sought by primary care. |
| 18. A rheumatology service should be supported in undertaking health equity assessments, using tools such as the Health Equity Assessment Tool, at appropriate time intervals. |
| 19. A rheumatology service should have a plan for implementing, and evaluating processes that aim to achieve equitable health outcomes for Māori, and other priority groups, as appropriate. |
| 20. Patients under the care of a rheumatology service should be offered telephone or video follow-up consultations, providing it is clinically appropriate to do so. |
| 21. Healthcare professionals providing care to patients with chronic rheumatic disease, admitted to a public (DHB) hospital, should be able to access inpatient review by a member of the rheumatology service that the patient's care falls under, if requested and clinically appropriate. |
| 22. Patients with chronic rheumatic disease who are clinically stable, and have a clear treatment plan, should be considered for discharge to primary care for ongoing follow-up without ongoing need for rheumatology service input (apart from administrative responsibilities, such as endorsement for methotrexate). |
